# Control effects of joint grouting and precision blasting on blasting damage in deep rock masses

**DOI:** 10.1038/s41598-025-22459-4

**Published:** 2025-11-05

**Authors:** Zihao Mao, Zidong Yue, Kaiwen Song, Yi Luo, Yangnuo Zeng, Mingju Mao, Xuan Zhang, Tingting Liu, Fuling Zeng

**Affiliations:** 1 School of Civil Engineering and Architecture, Wuhan University of Technology, Wuhan, China; 2 Guangxi Branch, China Construction Second Engineering Bureau Co., Ltd., Nanning, China; 3 Sanya Science and Education Innovation Park , Wuhan University of Technology , Sanya, China; 4 China Railway Design Corporation, Tianjin, China

**Keywords:** Blasting excavation, Jointed rock mass, Perimeter rock damage, Joint grouting, Fine blasting, Meizhou pumped storage power station, Engineering, Natural hazards, Solid Earth sciences

## Abstract

With the expansion of underground construction, deep rock mass excavation technology has attracted growing attention, as complex geology and developed joints in deep rock masses challenge rock mass integrity and stability. This paper, based on blasting excavation at Meizhou Pumped Storage Power Station, studies cavern displacement and surrounding rock damage from blasting in jointed rock masses via on-site vibration tests and ultrasonic experiments. Joints significantly increase blasting vibration velocity and rock damage depth. Two control measures are proposed: joint grouting and precision blasting, validated by numerical simulation. Joint grouting enhances joint stiffness, reducing unloading stress wave reflection and rock damage. Post-grouting, peak vibration velocities on both cavern sides decrease, more notably on the joint side, with reductions of 3.05 cm/s, 2.86 cm/s, and 3.97 cm/s in three directions. The right-side damage depth is reduced by 1.01 m, 40%. Precision blasting optimizes borehole layout and initiation networks, extending unloading time and reducing transient unloading effects. Its three-directional peak vibration velocity reductions are 2.53 cm/s, 2.53 cm/s, and 3.07 cm/s; non-joint side reductions are 1.85 cm/s, 1.53 cm/s, and 2.03 cm/s. Average damage depth decreases by 0.47 m (left) and 0.61 m (right).

## Introduction

With the deepening of underground engineering construction, the excavation technology of deep rock bodies is getting more and more attention from the engineering community^1^. Especially in the construction of hydropower projects, mine development, traffic tunnels and other underground facilities, blasting excavation is widely used as an economical and effective means for rapid excavation of rock bodies^2^. However, the geological environment of deep rock bodies is complex and varied, with developed joints, and the integrity and stability of rock bodies are significantly affected by blasting operations. The dynamic load generated by blasting not only leads to the displacement of the rock body around the cavern, but also may trigger the damage of the surrounding rock, which in turn affects the safety and stability of the underground project.

The discontinuity characteristics of rock mass are one of the key factors that must be considered in deep underground engineering. The presence of discontinuities significantly differs from the mechanical behavior of continuous medium materials, with their strength, deformation characteristics, and failure modes all controlled by the discontinuity surfaces. L. Liu^3^ used Digital Image Correlation (DIC) technology to monitor the full-field stress during the uniaxial compression process of prefabricated jointed rock samples with different dip angles and found that initial damage severely weakened the mechanical properties of the samples and caused significant stress concentration effects. Xie Heping^4^ discussed the intrinsic relationship between energy evolution during rock failure and the strength and overall failure of rocks, with results indicating that rock failure is the result of the combined action of energy release and dissipation. Pyrak-Noltel^5^ and Cook^6^ derived analytical solutions for the transmission and reflection of stress waves passing through a single discontinuity at any angle through theoretical analysis. Gong^7^ explored the mechanical characteristics and failure mechanisms of rock shear fracture under dynamic disturbance, the laboratory shear test and numerical simulation based on particle flow code (PFC) were carried out. Zhao^8,9^ combined the method of characteristics and the theory of displacement discontinuity to explore the transmission and reflection rules of stress waves passing through a set of parallel discontinuities. In actual rock masses, discontinuities often contain some fillings, and the low strength and large deformation characteristics of the fillings in discontinuity rock masses will change the mechanical properties of the structural surfaces. Zhu^10^ used a viscoelastic model to study the thickness issue of filled discontinuities. Gong^11^ aims to investigate the shear fracture behavior and failure precursors of intact granite and granite containing discontinuous joints under combined normal static loads and dynamic disturbances through shear fracture testing. Li^12^ studied the impact of different filled discontinuity thicknesses on the propagation rules of stress waves based on the linear elastic thin-layer model. In terms of numerical simulation, Zhao Jian^13^ simulated the propagation rules of explosive stress waves in discontinuity rock masses using the discrete element software UDEC and AUTODYN-2D. Liu Libo^14^ based on the assumption of stress and displacement discontinuities, combined numerical calculations and parameter analysis to explore the relationship between incident wave parameters, dynamic properties of filled discontinuities, and transmission and reflection coefficients of discontinuity surfaces. Li Peng. ^15^ found that the existence of discontinuities would seriously hinder the propagation of explosive stress waves, and both the peak velocity and propagation rate are significantly affected. Wang Shumin^16^ introduced the Barlow-Thompson model as a displacement discontinuity condition and derived the propagation equation of stress waves through a set of parallel viscoelastic discontinuities based on the time-domain recursive method. Chai Shaobo^17^, based on the time-domain recursive analysis method of stress wave propagation in discontinuity rock masses, introduced the propagation quality factor of waves and derived the propagation equation of P-waves in viscoelastic filled discontinuity rock masses considering in-situ stress conditions, and conducted parameter analysis on transmission and reflection coefficients and energy coefficients. Zhang Xianshang^18^ compared and analyzed the dynamic expansion process of cracks and impact failure modes, and studied the variation rules of stress intensity factors and energy release rates during the dynamic expansion process of Mode I cracks through experimental-numerical methods.

The control of displacement and damage around the cavern is key to ensuring the safety of underground engineering. Displacement of the rock mass around the cavern after blasting excavation may lead to excessive deformation or even destruction of support structures, while damage to the surrounding rock may cause further instability of the rock mass. Therefore, systematic research on the displacement and damage around the cavern caused by blasting excavation in deep discontinuity rock masses is of great significance for optimizing blasting design and improving the safety of underground engineering. Abuov^19^ and Carter^20^ found that the transient unloading of ground stress will excite dynamic unloading waves in the surrounding rock that are higher than the redistributed stress, which may cause tensile or shear damage to the rock mass outside the excavation profile; Yu^21^ found through indoor experiments that the dynamic stress redistribution caused by transient unloading will cause significant vibration response in the cavern surrounding rock, leading to excavation damage to the rock mass; through numerical simulation, Li^22,23^ believe that unloading damage will increase with the increase of ground stress levels; Zhu^24^ research shows that extending the stress release time can effectively reduce rock excavation damage; Lu^25^ and Yan^26^ compared the contribution of blasting impact and ground stress dynamic unloading to the surrounding rock damage through simulation calculations, and found that when the original rock stress is high or the excavation radius is large, the transient unloading effect will induce more severe rock mass damage than blasting impact.

The above scholars have conducted research on the mechanism of stress wave propagation in discontinuity rock masses and the control of displacement and damage around the cavern, However, there is relatively little research on specific measures for controlling displacement and damage in jointed surrounding rock; therefore, this paper takes the blasting excavation of Meizhou Pumped Storage Power Station as the engineering background, proposing two methods—joint grouting and precision blasting—to control the damage caused by blasting excavation in deep jointed rock masses, and validates these approaches through numerical simulation.

## Project overview

### Overview of geological conditions at Meizhou pumped storage power station

**Fig. 1:**
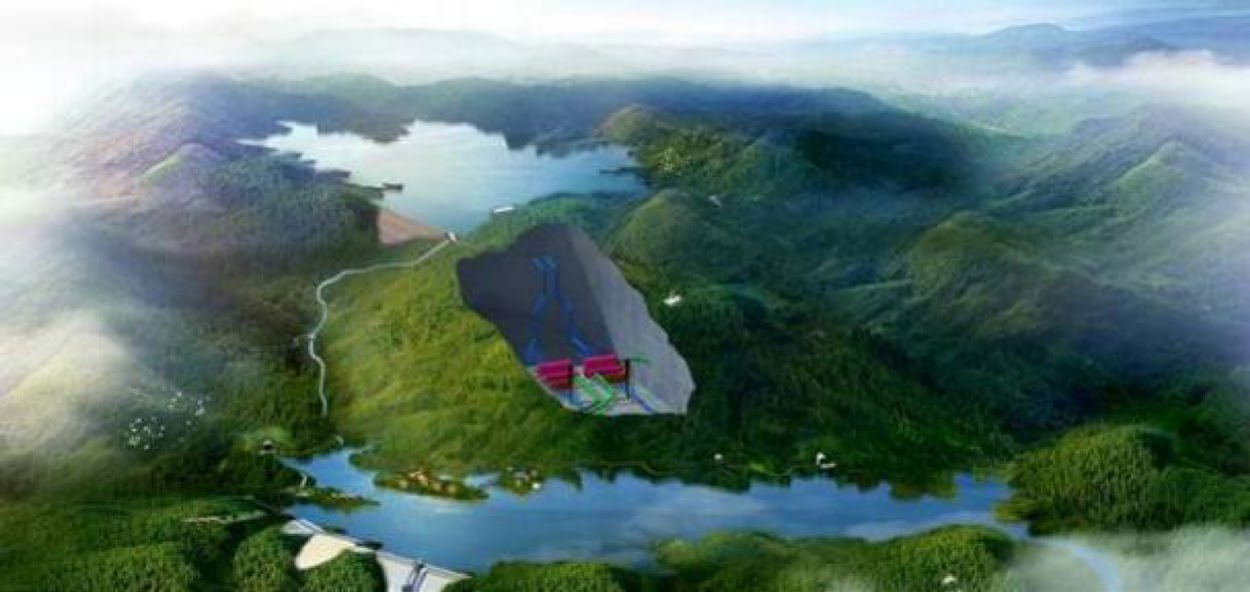
Effective diagram of Meizhou pumped storage power station.

The Meizhou Pumped Storage Power Station layout area has strong mountains, with the overall topography high in the southeast and low in the northwest.Fig. 1 shows a schematic diagram of the Meizhou Pumped Storage Power Station Mainly Yanshan period medium-grained black mica granite, only the upper and lower reservoir inlet/outlet open excavation section metamorphic rocks. The granite distribution area of the upper reservoir inlet/outlet is mostly exposed bedrock, with a small amount of residual slope deposits locally, which is 0.5 m ~ 1.5 m thick; the metamorphic distribution area is generally covered by the Quaternary System, and the residual slope deposits are 1 m ~ 2 m thick; the granite distribution area of the lower reservoir inlet/outlet is locally (at the bottom of the gully) exposed bedrock, with the residual slope deposits on both sides of the hillside being 1.0 m ~ 2.5 m thick, and the metamorphic distribution area is generally covered by the residual slope deposits which is 2 m ~ 3 m thick, and the metamorphic distribution area is generally covered by the residual slope deposits. The residual slope deposits are generally covered by 2 m ~ 3 m thick in the metamorphic rock distribution area, and the river alluvial deposits are distributed in the reservoir basin, which are composed of powdery sandy soil. Generally speaking, the granite rocks in the weakly weathered zone and the following areas are dense and hard, the integrity of the rock body is good, and the longitudinal wave velocity (Vp) of the rock body is generally greater than 5000 m/s. The granite rocks in the weakly weathered zone and the following areas are dense and hard, and the integrity of the rock body is good.

The intrusive contact surface of the granite body passes through the inlet and outlet of the upper and lower reservoirs respectively, and the contact surface is irregular, inclined to the surrounding rocks in the reservoirs, with a dipping angle of 25 ~ 30°, and the metamorphic rocks are in close contact with the granite. The metamorphic rocks have joints and fissures, the rocks are broken, the integrity of the granite body is good, and the faults and veins along the tail water hole are relatively developed.

**Fig. 2:**
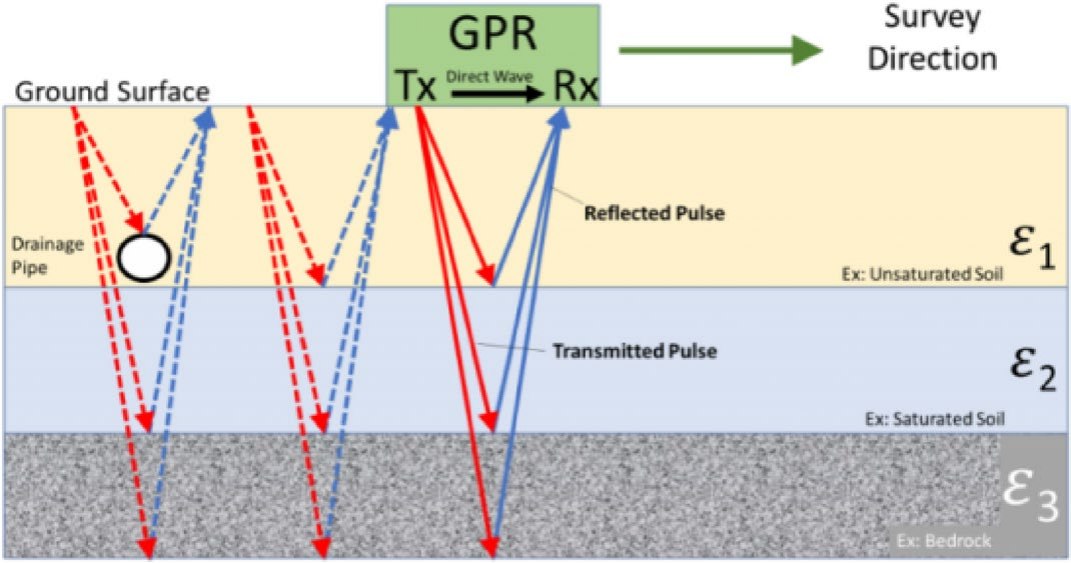
Schematic diagram of the principle of geological radar.

Based on the key information provided in the preliminary geological exploration report, the geological structure of the sidewalls of the cave chamber is obvious, and the sidewalls are detected by using geo-radar, which is a non-destructive, high-resolution geophysical probe that can penetrate the ground surface and reveal the underground structure, including rock layers, cracks, cavities and other geological features. This is essential for assessing the stability of the sidewalls and predicting potential geotechnical problems, the principle of which is shown schematically in Fig. 2.

According to the geological radar detection results and data, the top burial depth at the junction of the high-voltage cable shaft and the main transformer cave is about 440 m. The surrounding rocks of the cave chamber are mainly slightly weathered ~ fresh granite. There are small-scale faults and diorite veins, F41 passes near this location, the fault is 35/SW∠87°, the width of the broken zone is 0.05 m ~ 0.10 m, filled with veins, fault mud, poorly cemented or not cemented. The fault is well connected and highly permeable, and according to the engineering ground investigation data, the water injection test results at this location show that the permeability is more than 40%.

### Physical and mechanical properties of rocks (bodies) and geostresses

#### Indoor rock physical and mechanical property tests

In order to understand the physical and mechanical properties of the rock, a total of several sets of rock samples were taken at different locations and depths for testing. The granite in the project area is dense and hard with high strength. The test reflects that the regularity of the physical and mechanical properties of granite rocks is good, the average values of saturated compressive strength of weakly weathered and slightly weathered rocks are 70.4 MPa and 107.3 MPa respectively; the average values of static elasticity modulus are 42.1 GPa and 48.3 GPa respectively; the average values of softening coefficient are 0.70 and 0.72; and the average values of Poisson’s ratio are 0.24 and 0.22 respectively.

Sedimentary Rocks After metamorphism of rocks with different lithology, the physical and mechanical indexes of rocks are close to each other. Due to the fragmentation of metamorphic rocks, only in the slightly weathered rocks to obtain specimens, the statistical results are as follows: the average value of saturated compressive strength of rock is 83.5 MPa ~ 89.7 MPa; the average value of static elasticity modulus is 48.8 GPa ~ 66.5 GPa; the average value of softening coefficient is 0.65 ~ 0.79; and the average value of Poisson’s ratio is 0.22 ~ 0.23.

The results of the indoor shear test show that: The rock shear strength values are high, the f’ and c’ values of medium weathered rock are 1.79 and 4.86 MPa respectively, the f’ and c’ values of slightly weathered ~ fresh rock are 1.86 and 5.37 MPa respectively; the f’ and c’ values of the contact surface between medium weathered rock and concrete are 1.35 and 1.42 MPa respectively. the f’ and c’ values of slightly weathered ~ fresh rock in contact with concrete are 1.41 ~ 1.48 and 1.36 MPa ~ 2.05 MPa, respectively.

#### Field deformation test of rock mass

The field deformation test indexes of granite rock body have good regularity and objectively reflect the elasticity and deformation characteristics of the rock. The deformation modulus and elasticity modulus of the fresh and more intact granite (Class II perimeter rock) reached 10.41 GPa ~ 18.84 GPa and 15.78 GPa ~ 41.94 GPa; the values of deformation modulus and elasticity modulus of the granite (Class III perimeter rock) were 8.76 GPa and 14.52 GPa respectively.

#### Geostress test

Through the on-site ground stress test, the maximum principal stress near the underground plant is between 25.14 MPa ~ 28.33 MPa, the inclination angle is between 0.30°~37.09°, the azimuth angle is between 328.15°~28.90°, and it oscillates around 0°; the intermediate principal stress value is between 18.95 MPa ~ 23.80 MPa, the inclination angle is between 8.62°~43.39°, and the minimum principal stress value is between 9.05 MPa ~ 14.06 MPa, the inclination angle is between 50.69°~65.95°. 43.39°, and the azimuth angle is between 334.74° and 65.95°; the minimum principal stress value is between 9.05 MPa and 14.06 MPa, the inclination angle is between 50.69° and 72.92°, and the azimuth angle is between 315.52° and 82.03°.

## On-site blasting tests

### Overview of the construction of the secondary expansion blasting for the high-voltage cable shafts

**Fig. 3:**
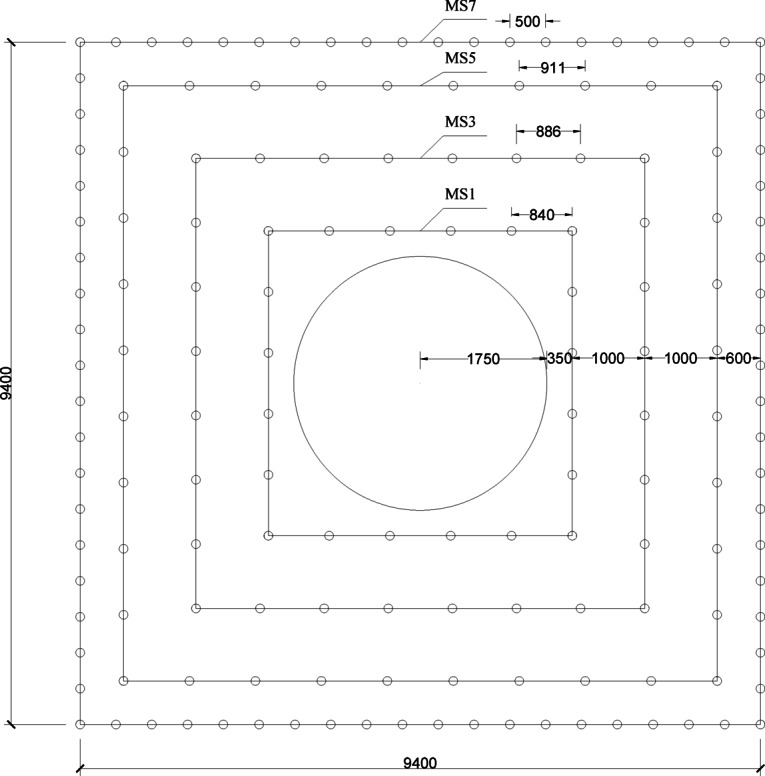
Layout and numbering of blast holes for the secondary expansion blasting of the high-voltage cable shaft.

The high-voltage cable shaft consists of three parts: the upper and lower shafts and the flat hole, the upper cable shaft is connected with the switching station, and the lower shaft is connected with the main transformer hole. The length of the upper shaft of the high-voltage cable is 250.77 m, the length of the lower shaft section is 135.3 m, and the section of the shaft is rectangular 11.15 m×10.8 m (large section of the wellhead section) and 9.35 m×9 m (standard section). The excavation of the high-voltage cable shaft adopts the excavation method of drilling the vertical guide shaft first, and then carrying out the expansion blasting to dig out the remaining part of the rock body. At this time, the excavation of the upper shaft and the flat hole has been completed, the main change hole has been opened to the bottom of the lower shaft, and has completed the excavation of the lower shaft in the guide shaft and the first expansion blasting, will soon be carried out from top to bottom of the second expansion blasting of the last through the last blasting, and the main change hole connected.

High-voltage cable shaft expansion secondary excavation blasting blasting excavation section area of a total of about 82.03 m2, 160 drill holes, blasting volume of 246.1 m3, the total charge of 226.80 kg, the explosives unit consumption of 0.92 kg/m3. Specific design dimensions and the distribution of holes, such as shown in Fig. 3.

**Fig. 4:**
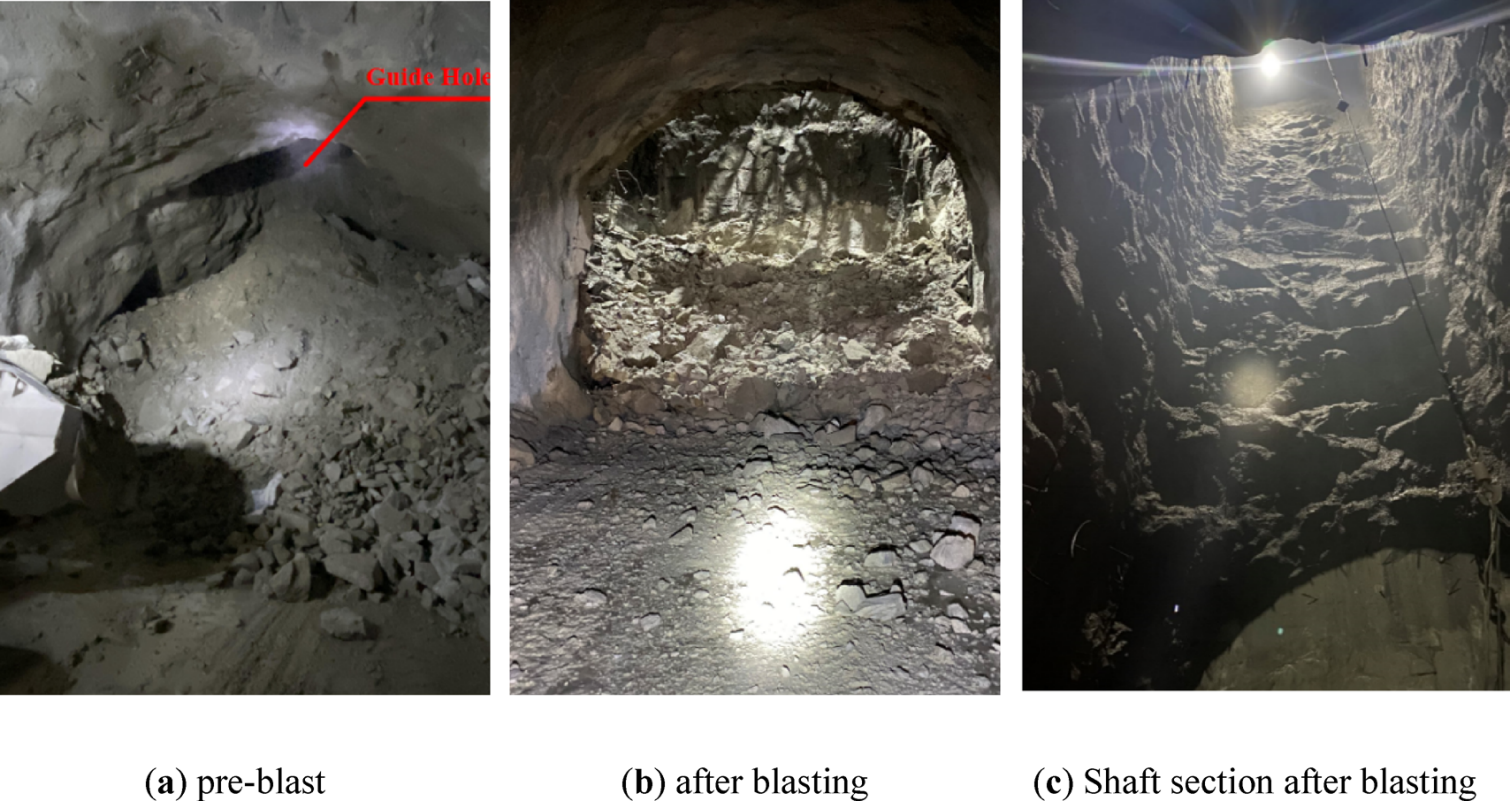
Photographs of the site of the secondary expansion blasting of the high-voltage cable shaft.

Field drilling and charging parameters are shown in Table 1, and the project site photographs are shown in Fig. 4.

**Table 1:** Drilling and charging parameters for secondary expansion blasting site for high voltage cable Shafts.

Blast hole	Detonator segments	Borehole Diameter (mm)	Borehole Depth (cm)	borehole spacing (mm)	Charge Length (cm)	Single Hole Charge Quantity (kg)	Linear Density (kg/m)
Breaking Hole	MS1	42	350	840	280	2.1	0.6
	MS3	42	350	886	280	2.1	0.6
	MS5	42	350	911	280	2.1	0.6
Peripheral Hole	MS7	42	350	500	280	0.7	0.2

### Experimental programme design

As the expansion blasting above the shaft has been completed, the ground stress at the shaft wall is supported by the last layer of rock to be excavated, which will release a large amount of energy along with the strong transient unloading of the ground stress. Moreover, according to the site investigation report, there is a fault (F41) with a width of 0.05 m ~ 0.10 m, which is approximately parallel to the cave wall at a depth of about 14.7 m to 16.4 m in the right side of the main changeable cave wall, which is filled with rock veins, fault mud, and has the characteristics of good connectivity and strong water permeability. According to the previous study, it can be assumed that this joint belongs to the long joints with close distance (D ≈ 2.2a), and its joint stiffness is obviously low, which may bring serious damage and deformation to the surrounding rock under the transient unloading action. Therefore, on-site monitoring of the vibration velocity of the mass and acoustic testing of the depth of damage to the surrounding rock were carried out for the last penetration blasting in the second expansion blasting of the high-voltage cable shaft.

#### Blast vibration test

**Fig. 5:**
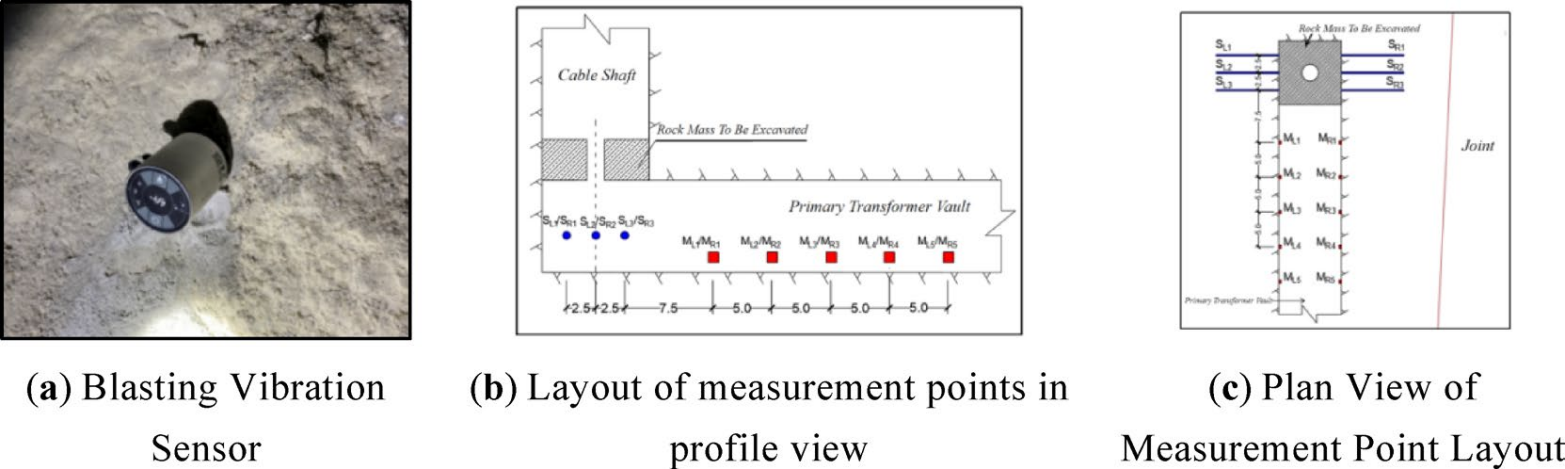
Arrangement of Blast Vibration Sensors.

The ISensor-ISV-316 Intelligent Measurement Terminal produced by Sichuan Top Measurement and Control Technology Co., Ltd. is used as the blasting vibration sensor. The intelligent sensor is a compact cylinder with a height of 48 mm and a diameter of 42 mm, which can independently complete the three-way vibration velocity measurement with strong reliability and stability, and has built-in numerical processing and storage functions. And with wifi communication module, can achieve on-site network control and data transmission, can maximise the simplification of the test site workflow. Blast vibration sensor field installation shown in Fig. 5 (a), by drilling holes in the rock wall, and the bottom of the ground rod embedded in the drilled holes so that the instrument and the rock solidification. When fixing the device, it was mounted as horizontally as possible (X and Y in the same horizontal plane), with Y pointing horizontally towards the centre of the blast.

Considering the safety of experimental personnel and experimental instruments, the blasting vibration test points are arranged on the left and right sides of the main change hole at a horizontal distance of 10 m from the shaft, at a height of about 1 m from the ground, and then two left and right points are set up at intervals of 5 m along the axial direction of the main change hole room, with a total of 10 points, numbered ML1 ~ ML5 and MR1 ~ MR5 respectively, as shown in Fig. 5(b) and (c).

#### Perimeter rock damage depth acoustic test

In order to get the damage condition of the surrounding rock, the damage depth sonic test test was carried out on the rock body near the blasting site. The sonic test holes were arranged below the rock body to be excavated, with three test holes on the left and right walls, and a total of six sonic test holes, numbered SL1 ~ SL3 and SR1 ~ SR3, respectively, and the specific layout is shown in Figs. 5(b) and (c). The single-hole acoustic wave test method was adopted in the test. At each test point, one test hole with a depth of about 9 m and a borehole diameter of 65 mm was arranged, and in order to ensure a good coupling effect during the test, the test holes were filled with water, so that all the holes were slightly inclined downwards. One test was carried out before and after the blast, and the acoustic data were recorded before and after the blast. After the completion of the pre-blast test, in order to prevent debris from falling into the hole caused by clogging, all holes need to be covered with sandbags. When the site blasting is completed, quickly clean up the scattered debris and remove the sandbags, and then start the post-blast acoustic wave test. The two measured sound wave velocity in the surrounding rock for comparison, you can calculate the degree of damage to the surrounding rock caused by blasting.

**Fig. 6:**
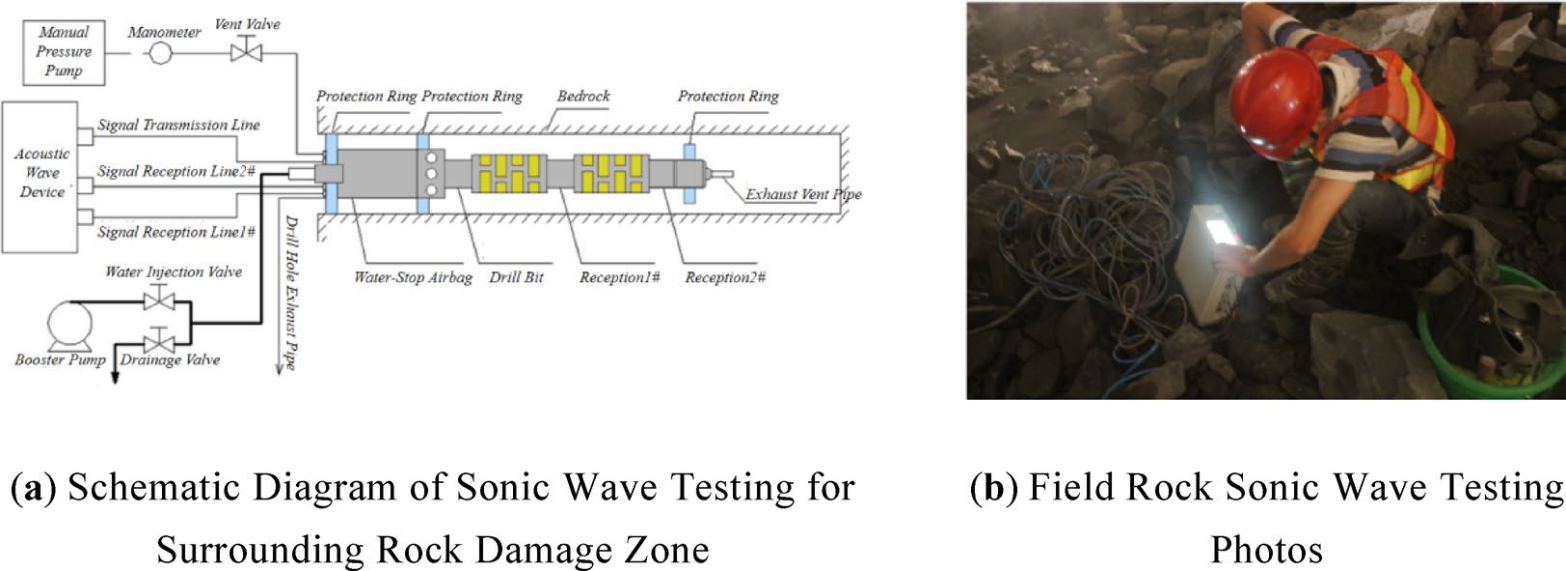
Perimeter rock damage depth acoustic test.

A schematic diagram of the on-site acoustic testing device is shown in Fig. 6(a), and a photo of the site is shown in Fig. 6(b). In the acoustic wave testing process, a bottom-to-borehole testing method was adopted, in which the acoustic wave probe was first placed at the bottom of the borehole to test the acoustic wave velocity of the nearby rock mass, after which the instrument was withdrawn outwards and the wave velocity was tested at intervals of 0.2 m, and when the difference in the acoustic wave velocity between the two neighbouring measurement points was greater than 20%, it was considered that obvious damage had occurred in the rock mass at that location .

### Test results

#### Blast vibration velocity test

The blasting vibration velocity was monitored on site by a blasting vibration tester and the peak vibration velocities were extracted in three directions: vertical (X) direction, horizontal parallel to the hole axis (Y) direction, and horizontal perpendicular to the hole axis (Z) direction.

**Fig. 7:**
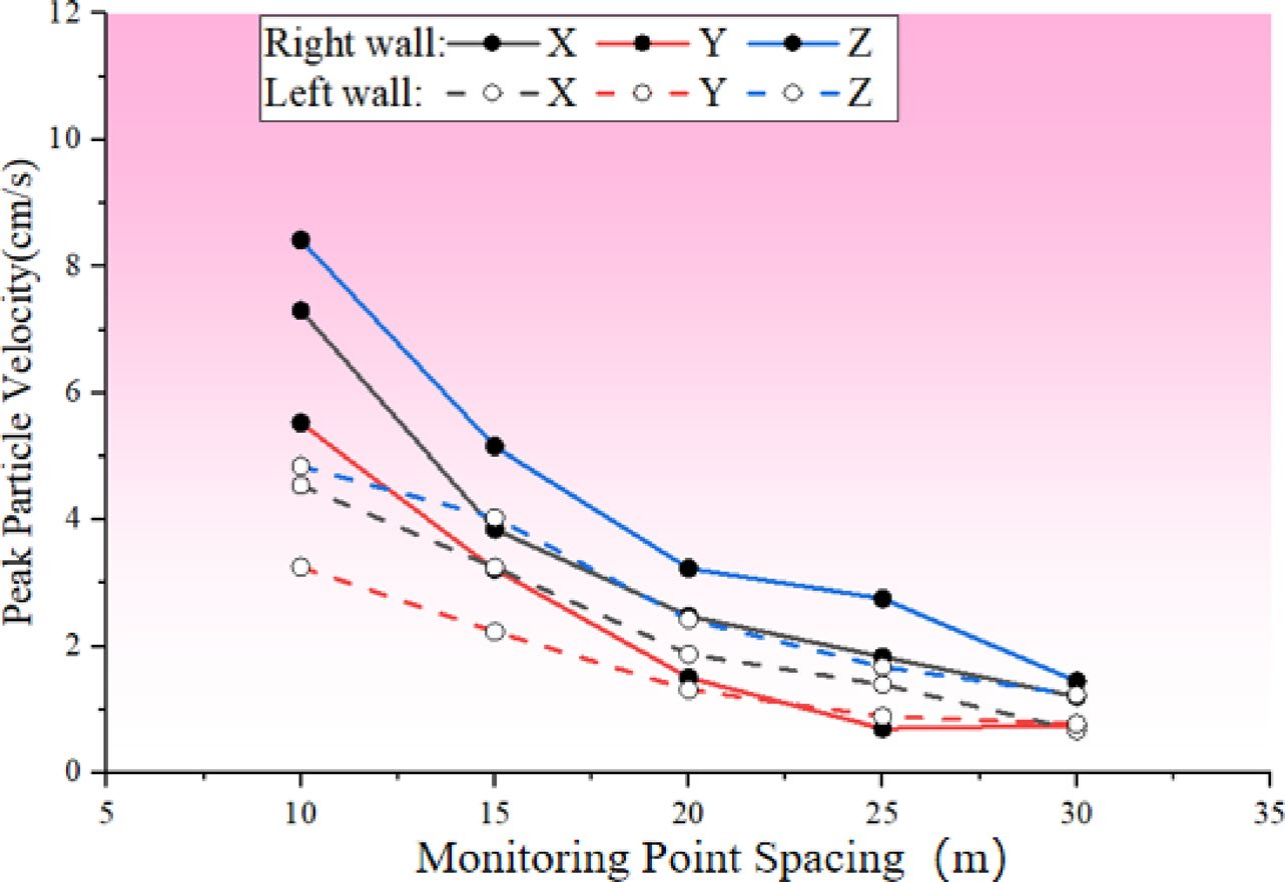
Peak vibration velocities at each measurement point on the left and right walls of the chamber.

As shown in Fig. 7，Comparison of different directions under the left wall and the right wall of the measurement point peak velocity can be found, with the change in the distance from the measurement point, the trend of the peak velocity change in each direction is generally similar, but due to the existence of the right wall of the main change in the direction of the wall of the cave is approximately parallel to the wall of faults, the reflection of the role of the right wall of the blast vibration velocity is obviously higher than the same distance from the left side of the wall; and the closer the measurement point and the source of the blast . The closer the distance between the measurement point and the source, the greater the difference between the peak velocity of the measurement point of the right wall and the left wall of the cave. Especially in the horizontal perpendicular to the direction of the hole axis (Z) direction, the right and left walls of the MR1 and ML1 measurement points between the peak velocity difference reached 3.55 cm/s.

#### Perimeter rock damage depth acoustic testing

**Fig. 8:**
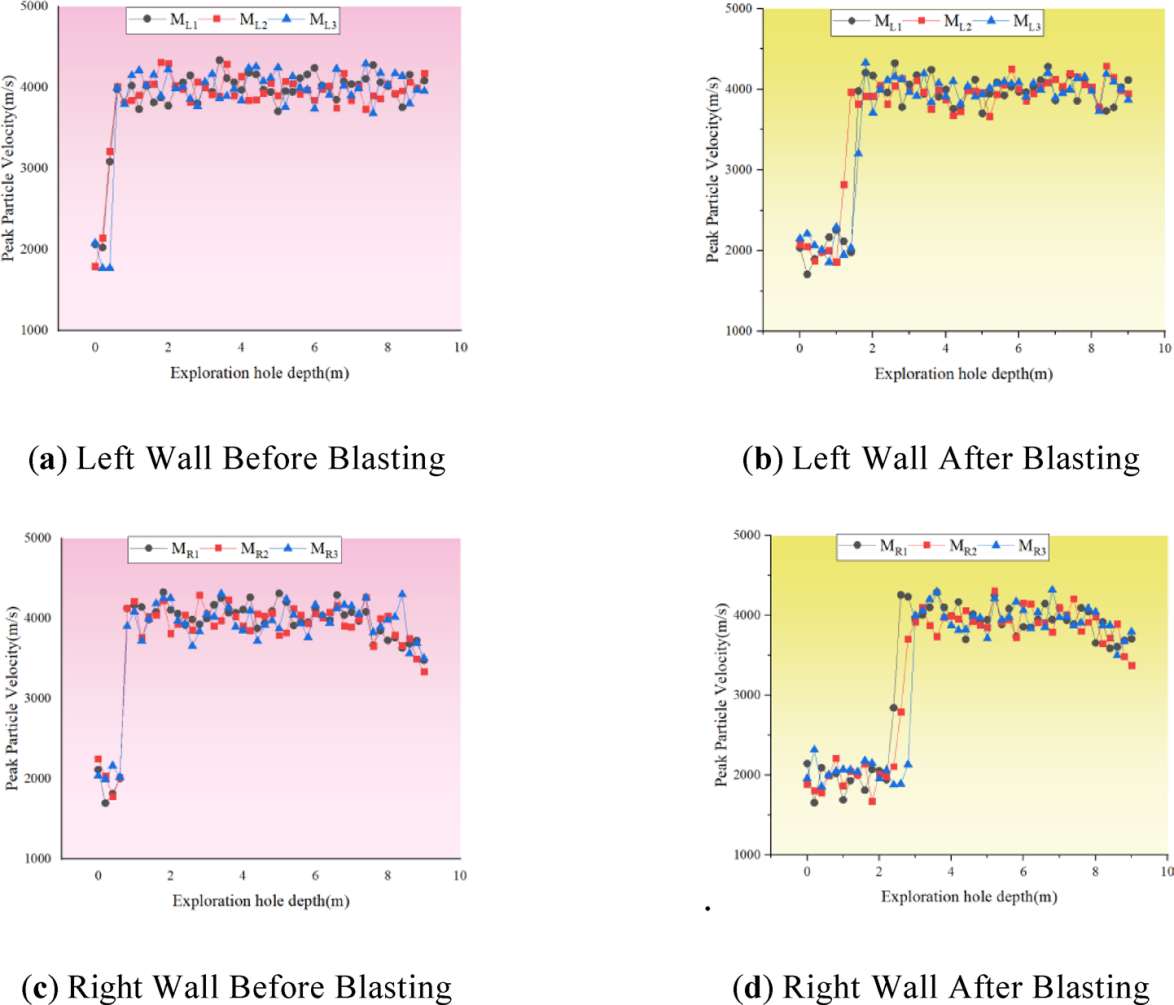
Acoustic test data before and after blasting.

Field were carried out in the main change hole left wall and the right wall of the rock body before and after the explosion acoustic wave test, the acoustic wave test results of different measurement points shown in Fig. 8. From the Fig. 8, it can be seen that in the high-voltage cable shaft expansion before the blasting, the acoustic wave velocity in the rock body closer to the critical surface of each measurement point is about half of that in the deeper rock body, the former is about 2000 m/s, while the latter has reached about 4000 m/s. Therefore, it can be assumed that in the previous blasting and excavation process, the surrounding rock has been damaged to a certain extent under the action of explosive power. And after the completion of the shaft expansion blasting, the wave velocity drop of different degrees occurred in each measuring hole near the drilling hole, indicating that the blasting excavation of the shaft does have a certain degree of damage effect on the rock body of the cave wall.

**Fig. 9:**
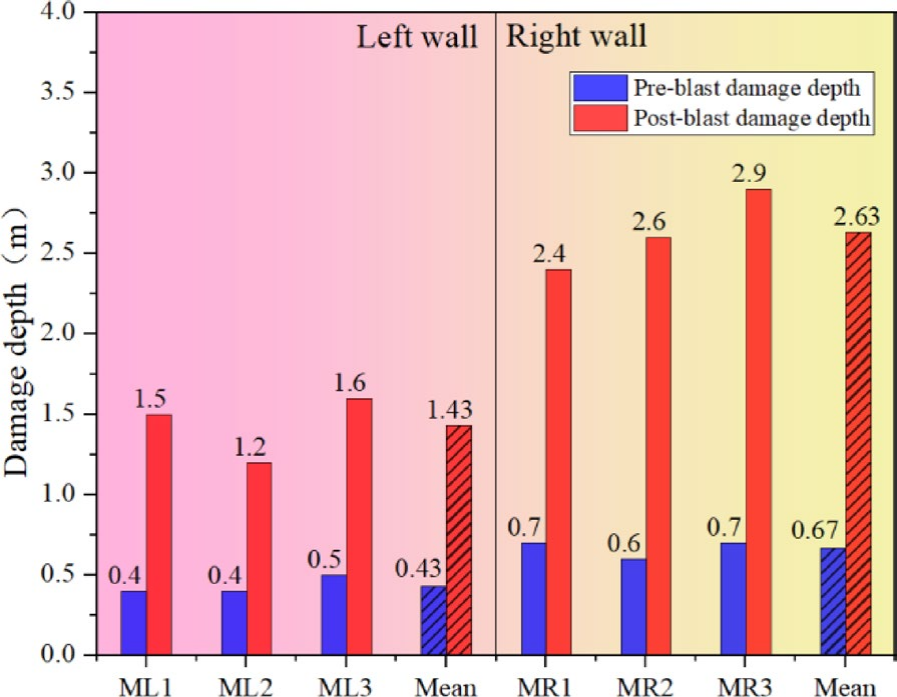
Comparison of the depth of damage to the surrounding rock before and after blasting at different measurement points.

In order to see more clearly and intuitively the influence of shaft blasting and excavation on the damage depth of the surrounding rock, the average value of the damage depth of the rock body deduced from the change value of acoustic wave velocity before and after the blasting and excavation is shown in Fig. 9, which shows that due to the existence of the internal joints in the right wall of the cavern chamber, the damage depth of the surrounding rock is obviously greater under the joint action of blasting loading and transient unloading. After blasting and excavation, the average damage depth of the left wall is 1.43 m, while the average damage depth of the right wall reaches 2.63 m, which is about 1.0 m or so more than the damage depth of the left wall.

## Modelling and validation

### Numerical modelling

**Fig. 10:**
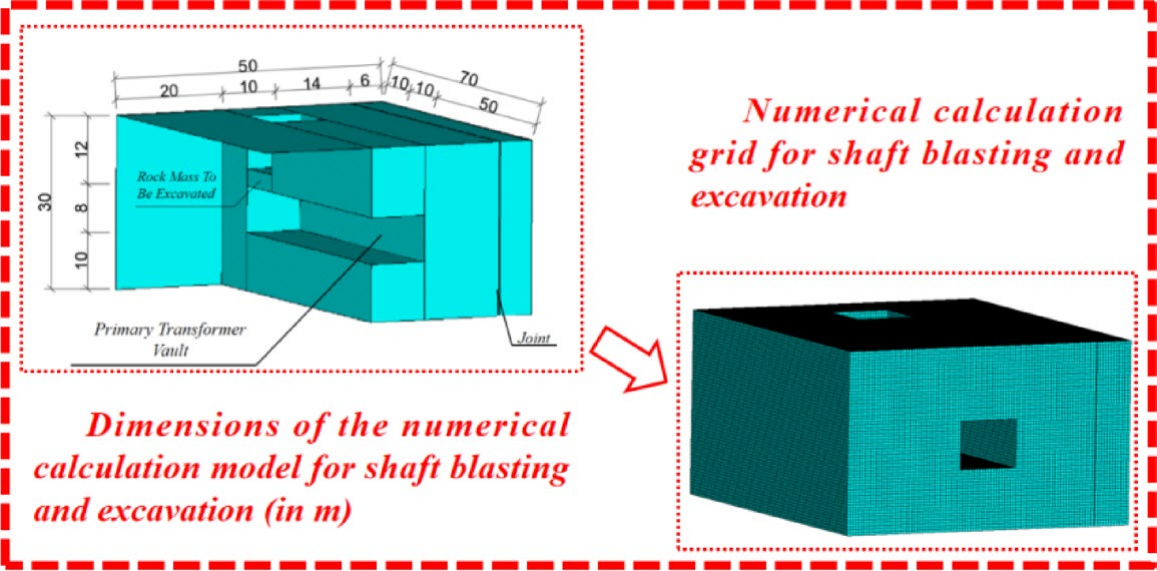
Schematic diagram of the numerical simulation model.

Using the Meizhou pumped storage power station shaft blasting excavation project as a case study, along with test data, numerical simulations were conducted using the numerical computation software LS-DYNA. Given the large scale of the model, the unit system of kg-m-s was employed in the modeling process, with the overall dimensions of the model set at 50 m by 70 m by 30 m. The cell side length was set to 0.5 m, resulting in approximately 630,000 cells. The dimensions of the shaft were aligned with field data, representing a rectangular cavity of 10 m by 10 m. Concurrently, since the main variable cave was not the primary object of study, it was simplified to a rectangular cavity of 8 m by 10 m. The model was constructed based on data from the engineering ground investigation. The investigation revealed the presence of the F41 fault near the site, characterized by a fracture zone width of 0.05 m to 0.10 m, filled with rock veins and fault gouge, which are either poorly cemented or uncemented, exhibiting good connectivity and strong water permeability. The water permeability, as indicated by the water injection test, exceeds 40, classifying it as a typical weak joint. Consequently, a vertical joint was modeled at a depth of 14 m from the right wall of the main variable cave, with a joint stiffness of kn = 0.05E, determined based on engineering experience^27^. he joint’s calculation was performed using the face-to-face contact algorithm with a penalty function. The specific model schematic and Mesh Splitting Diagram are depicted in Fig. 10.

The numerical model dimensions (50 m × 70 m × 30 m) are deemed rational for the study’s scope and objectives. Given the dominant blasting-induced stress wavelengths in granite (λ ≈ 25–33 m for f = 150–200 Hz), the model’s minimum dimension (30 m) satisfies the criterion L ≥ λ, ensuring accurate resolution of high-frequency stress wave propagation. While low-frequency waves (λ > 100 m) may not be fully captured, the non-reflective boundary conditions effectively suppress artificial reflections, and the spatial resolution (0.5 m cell size) sufficiently resolves critical high-frequency dynamics. The model’s simplification aligns with engineering practicality, focusing on key features (e.g., F41 fault) and transient unloading effects. Overall, the setup adequately supports the investigation of blasting-induced vibration and damage mechanisms within the targeted frequency range, validating its applicability to the Meizhou case study.

While the average in-situ stress values provide a representative baseline for simulation, the potential influence of stress variability was further investigated. A series of sensitivity analyses were conducted by varying the magnitudes of the three principal stresses within their measured ranges (i.e., ± 10% from the average values). The results demonstrated that such variations induced changes of less than 6% in the predicted peak particle velocities and less than 0.15 m in the damage depth. This indicates that while stress uncertainty does have an effect, the overall trends and comparative conclusions regarding the effectiveness of grouting and precision blasting remain robust and valid.

Based on the on-site ground stress test data, the tectonic stress predominates near the underground plant area. The maximum principal stress in this region ranges from 25.14 MPa to 28.33 MPa, with an average value of 26.7 MPa used in numerical calculations. This stress is aligned with the axis of the main transformer hole and is applied as the initial load on the front surface boundary of the model. The intermediate principal stress ranges from 18.95 MPa to 23.80 MPa, with a mean value of 21.4 MPa considered in the vertical direction for numerical calculations, applied to the upper surface boundary of the model. The minimum principal stress value ranges from 9.05 MPa to 14.06 MPa, with a mean value of 11.5 MPa taken as the horizontal direction perpendicular to the axis direction of the main transformer hole in numerical calculations, applied to the right surface boundary of the model. Concurrently, displacement constraints perpendicular to the surface are applied at the corresponding rear, lower, and left boundaries. Furthermore, to mitigate the boundary effects of the model, reflection-free boundary conditions are applied to all model boundaries to prevent stress wave reflections.

The calculations employ the implicit-explicit-implicit sequential calculation method. Initially, rock units to be excavated are retained in the modeling process. The geostress equilibrium stage is calculated using the implicit method, from which the load distribution on the excavated surface is derived. Following this, the equivalent explosion load time course on the excavated surface is calculated according to Eq. (1). The expression for the time course of the equivalent blast load on the excavated surface is calculated as follows:

1 $$\:\frac{{\partial ^{{\text{2}}} {\text{u}}}}{{\partial {\text{t}}^{{\text{2}}} }}{\text{ = c}}^{{\text{2}}} \left( {\frac{{\partial ^{{\text{2}}} {\text{u}}}}{{\partial {\text{r}}^{{\text{2}}} }}{\text{ + }}\frac{{\text{1}}}{{\text{r}}}\frac{{\partial {\text{u}}}}{{\partial {\text{r}}}} - \frac{{\text{u}}}{{{\text{r}}^{{\text{2}}} }}} \right){\text{,r}} \ge {\text{a,t}}> {\text{0}}$$

This equation is the equation of motion of a mass based on the assumption of plane strain and was proposed by Timoshenko and Goodier in 1954^28^. Where u is the displacement of the plasma point in the axial direction; $\:\text{c}$ denotes the longitudinal wave velocity in the plane,$\:\text{c}=\sqrt{\frac{\text{E}{\rho}}{\text{1-}{{\upsilon}}^{\text{2}}}}$, where E is the modulus of elasticity of the material,${\upsilon}$ denotes Poisson’s ratio, and ${\rho}$ denotes the density of the material. In order to solve the problem, it is also necessary to describe its boundary conditions and initial conditions:

And Eq. (2) to calculate the transient unloading time history on the profile surface and apply it as an initial boundary condition on the excavation profile surface as the initial boundary condition for the explicit dynamic calculation.

The expression for the transient unloading time course on the contour surface is calculated as:

2 $$\:\sigma _{{\text{r}}} {\text{|}}_{{{\text{r = a}}}} {\text{ = }}\left\{ {\begin{array}{*{20}c} { - \frac{{{\text{p}}_{{\text{0}}} {\text{t}}}}{{{\text{t}}_{{\text{u}}} }},{\text{0}} < {\text{t}} \le {\text{t}}_{{\text{u}}} } \\ { - {\text{p}}_{{\text{0}}} {\text{,t}}_{{\text{u}}} \le {\text{t}}} \\ \end{array} } \right.$$

Subsequently, the analysis transitions to the explicit dynamic analysis calculation program to precisely analyze the dynamic process under the coupling effects of blasting load and transient unloading, thereby obtaining the vibration data of the surrounding rock. Ultimately, after a period of explicit calculation, the analysis switches to implicit calculation to rapidly determine the damage distribution pattern of the surrounding rock in its final stable equilibrium state.

The HJC constitutive model is one of the most widely utilized models for numerical simulations of rock blast excavation^29–31^ Proposed by Holmquist et al. in 1993, this model serves as a constitutive model for brittle materials like rock and concrete under conditions of large strains, high strain rates, and high pressures^32^. It acknowledges that rocks and concrete are not typically subjected to high stress states. The HJC model comprehensively considers the characteristics of brittle materials such as rock when impacted and exploded, including the effects of compression, multi-axial stress, strain rate, and tensile and compressive damage^33^.

In the model, equivalent strength is represented by the compressive effect, expressed through pressure, strain rate, and cumulative damage. Cumulative damage is calculated by considering plastic strain and the magnitude of pressure, while pressure accounts for the irreversible crushing effect and is derived from the calculation of instantaneous strain.

The expression for the HJC intensity model is:

3 $$\:\sigma ^{{\text{*}}} {\text{ = }}[{\text{A}}\left( {{\text{1}} - {\text{D}}} \right){\text{ + BBP}}^{{{\text{*N}}}} ]\left[ {{\text{1 + Cln}}\left( {\dot{\varepsilon }^{{\text{*}}} } \right)} \right]{\text{ = S}}_{{{\text{max}}}} ]$$

Where $\:{\sigma}^{{*}}$is the normalised equivalent stress, which is equal to the ratio of the actual equivalent stress and the uniaxial compressive strength; $\:{\text{S}}_{\text{max}}$is the maximum value of the normalised equivalent stress; $\:{{\varepsilon}}^{\text{*}}$is the normalised equivalent strain rate, which is equal to the ratio of the loaded strain force and the reference strain rate; D is the damage variable, and the rest of the A, B, C, and N are the material parameters, which characterise the magnitude of cohesion and the hardening property of the material, respectively.

The damage evolution equation for the HJC model is:

4 $$\:{\text{D = }}\sum \: \frac{{\Delta \varepsilon _{{\text{P}}} {\text{ + }}\Delta \upmu _{{\text{P}}} }}{{\varepsilon _{{\text{P}}}^{{\text{f}}} {\text{ + }}\upmu _{{\text{P}}}^{{\text{f}}} }}{\text{D = }}\sum \: \frac{{\Delta \varepsilon _{{\text{P}}} {\text{ + }}\Delta \upmu _{{\text{P}}} }}{{\varepsilon _{{\text{P}}}^{{\text{f}}} {\text{ + }}\upmu _{{\text{P}}}^{{\text{f}}} }}$$

5 $$\:\varepsilon _{{\text{P}}}^{{\text{f}}} {\text{ + }}\mu _{{\text{P}}}^{{\text{f}}} {\text{ = D}}_{{\text{1}}} \left( {{\text{P}}^{{\text{*}}} {\text{ + T}}^{{\text{*}}} } \right)^{{{\text{D}}_{{\text{2}}} }} \varepsilon _{{\text{P}}}^{{\text{f}}} {\text{ + }}\mu _{{\text{P}}}^{{\text{f}}} {\text{ = D}}_{{\text{1}}} \left( {{\text{P}}^{{\text{*}}} {\text{ + T}}^{{\text{*}}} } \right)^{{{\text{D}}_{{\text{2}}} }}$$

where ΔεP and ΔµP are the equivalent strain increment and plastic volume strain increment in one time step, and $\:{\varepsilon}_{\text{P}}^{\text{f}}$ and $\:{\mu}_{\text{P}}^{\text{f}}$ represent the equivalent plastic strain and plastic volume strain at the time of damage, respectively.

The specific material parameters are based on the data measured in the indoor tests, combined with the physico-mechanical parameters obtained from the fitting, and the values of the HJC model parameters for the rock specimens are shown in Table 2.

**Table 2:** Material parameters for numerical modelling of jointed rock mass.

Parameter name	Retrieve a value
Density *ρ*(kg/m)^3^	2950
Modulus of elasticity E (GPa)	48.3
Characterised viscous strength factor A	0.57
Characterised pressure hardening factor B	2.5
Strain rate factor C	0.0127
Pressure hardening index N	0.79
Quasi-static uniaxial compressive *strengthf*_C_ (GPa)	0.1073
Maximum tensile stress T (GPa) that the material can withstand	0.0128
Reference strain rate EPSI	0.00001
Minimum plastic strain at rupture of the material EFMIN	0.01
Dimensionless Maximum Strength SMAX	5
Hydrostatic pressure Pcrush (GPa)	0.015
Volumetric Strain Ucrush	0.0019
Longitudinal coordinates of the intersection of the fitted curves for the plastic and compact phases Plock (GPa)	1.66
Pressure limit volumetric strain Ulock	0.12
Damage parameter d1	0.024
Damage parameter d2	1
K1 (GPa)	12
K2 (GPa)	25
K3 (GPa)	42
Failure parameter FS	0.1

### Comparative validation of numerical calculations and field measured data

**Fig. 11:**
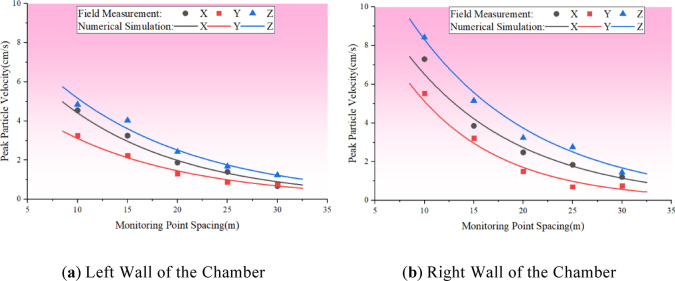
Comparison between numerical simulation and measured data of peak blasting velocity.

The peak vibration velocity curves from both sides of the main variable cave in the numerical model were extracted and compared with field-measured data, as depicted in Fig. 11. The Fig. 11 reveals a high degree of consistency between the blast vibration data from the numerical simulation and the field measurements. The simulation results are fundamentally in line with the measured data, with an average deviation of all measurement points being 0.22 cm/s and an average deviation rate of approximately 5.13%. The maximum deviation among the measurement points is observed at MR1, which is located 10 m from the right wall of the cave chamber, with a deviation value of 0.78 cm/s and a deviation rate of about 10.7%. Consequently, it can be concluded that the numerical simulation accurately and effectively captures the dynamic response of the surrounding rock, reflecting the actual engineering scenario.

The maximum deviation of 10.7% observed at measurement point MR1 between numerical simulations and field data may stem from the following factors: First, the simplified modeling of the F41 fault as a vertical joint with uniformly distributed fill materials (e.g., fault gouge and veins) fails to fully characterize local variations in the actual fracture zone width (0.05–0.10 m) and heterogeneity of fill properties, leading to inaccuracies in simulating stress wave reflection and transmission behaviors at the fault interface. Second, the equivalent joint stiffness (Kn = 0.05E) adopted in the model inadequately accounts for the energy dissipation effects caused by the fault’s high permeability (> 40 Lu), potentially underestimating the energy attenuation of dynamic unloading waves. Additionally, localized microfractures undetected by ground-penetrating radar or inherent anisotropy of the rock mass near the sensor installation site may introduce stochastic errors in field measurements. These modeling simplifications and geological complexities collectively contribute to the observed discrepancy at MR1.

**Fig. 12:**
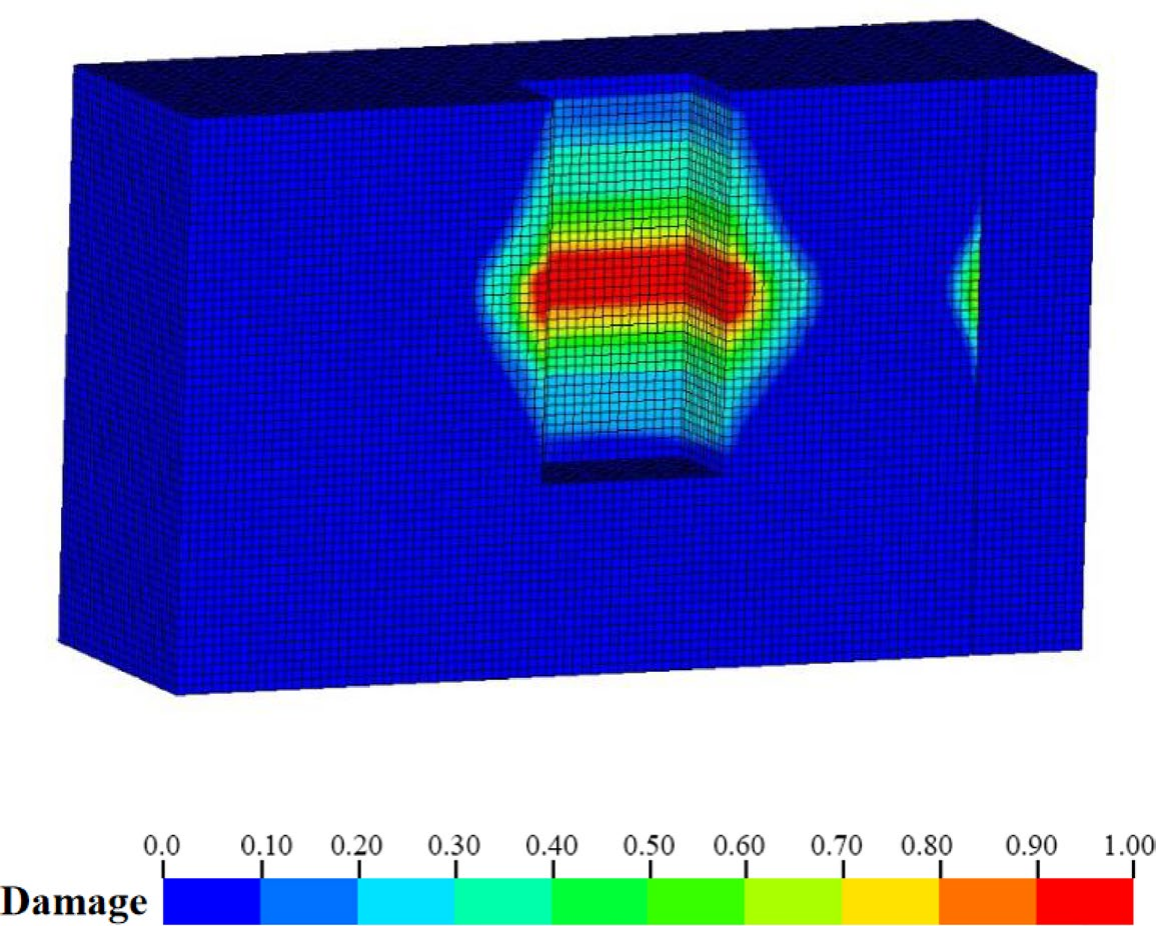
Damage distribution after extended blasting of cable shaft.

Figure 12 illustrates the damage distribution cloud map at the shaft cross-section derived from numerical simulation. The Fig. indicates that, under the combined influence of blasting load and transient unloading, there is a pronounced perimeter rock damage phenomenon in all directions adjacent to the excavation surface. Nevertheless, the presence of joints (F41) results in a significantly greater damage depth and extent on the right wall compared to the left wall. Additionally, the reflection damage zone attributable to transient unloading is observable within the joints.

**Fig. 13:**
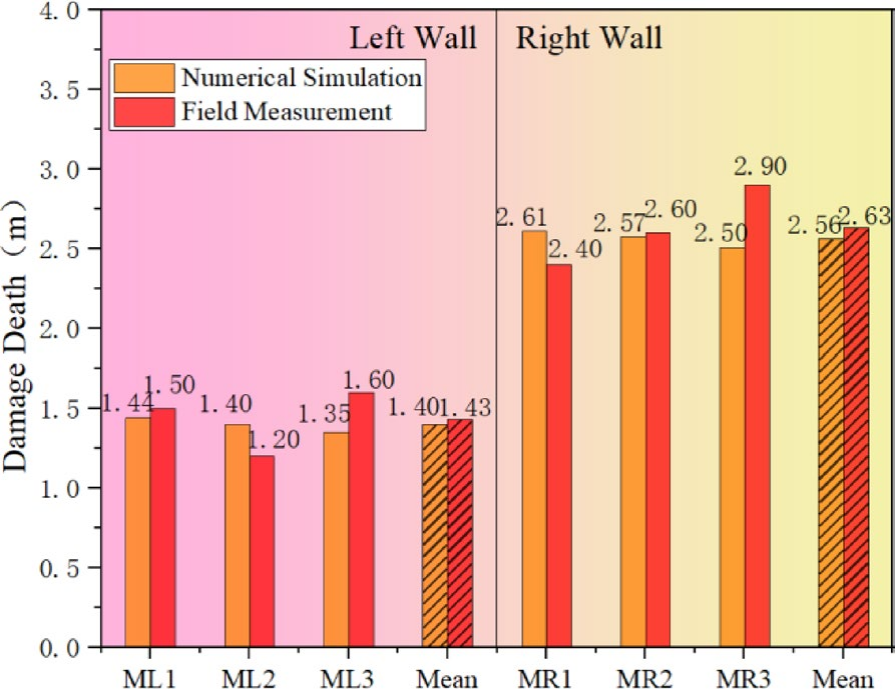
Comparison between numerical simulation and measured data of the depth of damage to the surrounding rock.

In the model, the segment corresponding to the actual acoustic measurement site in the field is selected, and the damage depth is extracted for comparison with the measured data as shown in Fig. 13. The Fig. 13 reveals that, due to the good homogeneity of the rock mass in the numerical simulation, the calculated damage depth is relatively uniform on the same side of the cavern wall. Overall, the damage depth of the surrounding rock derived from the numerical simulation and the acoustic tests at the project site are essentially consistent, with differences in the mean damage depths between the left and right walls being 0.03 m and 0.07 m, respectively, indicating a relatively small discrepancy. The mean depth differences of 0.03 m and 0.07 m are considered small errors. Thus, it can be concluded that numerical simulation is a viable method for studying the damage extent of surrounding rock under the dynamic disturbance of transient unloading during blasting excavation in this scenario.

### Deep jointed rock body blasting excavation perimeter rock damage control measures

Based on field-measured data and numerical simulation outcomes, it has been observed that the vibration velocity on the right wall of the cavern chamber and the damage depth within the surrounding rock are markedly increased when joints are present within the rock. To address this issue, two potential solutions have emerged from previous studies: one involves enhancing joint stiffness through grouting to diminish the reflection effect of unloading stress waves at the joints, thereby controlling the damage extent of the perimeter rock; the second solution is to optimize blasting parameters with the goal of extending the unloading duration and reducing the unloading rate, which can mitigate the dynamic effects of transient unloading and consequently decrease the damage range of the surrounding rock.

#### Nonlinear control mechanisms of grouted joint stiffness on Blasting-Induced deformation and damage

Based on the field and numerical findings that joints exacerbate blasting vibrations and damage, this section proposes and evaluates two targeted control measures: joint grouting and precision blasting. The mechanisms, numerical implementation, and effectiveness of each method are detailed in the following subsections.

##### Inhibitory effect of grouted joint stiffness on sidewall deformation

**Fig. 14:**
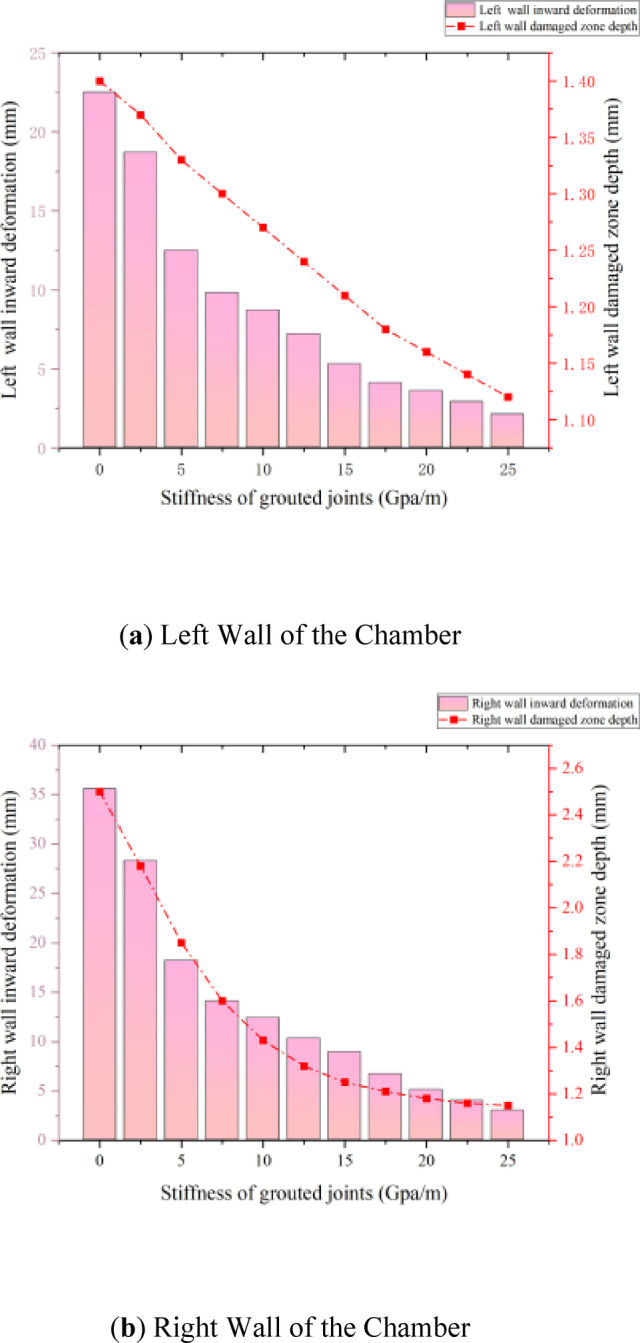
Effect of Grouted Joint Stiffness on Blasting-Induced Deformation of Side walls Explanation.

In blasting construction, the stability of sidewalls is directly governed by the stiffness characteristics of grouted joints. Based on the measured data analysis of the right wall (Fig. 14), when the grouted joint stiffness is incrementally increased from 0 GPa/m to 25 GPa/m, the inward deformation of the right wall decreases significantly from an initial value of 2.6 mm to 1.0 mm, representing a reduction of 61.5%. Although specific numerical values for the left wall are not explicitly provided, its deformation trend exhibits high consistency with the response pattern of the right wall (Fig. 1), demonstrating the universal applicability of stiffness in suppressing sidewall deformation. The essence of this phenomenon lies in the optimization of the mechanical properties of grouted joints: high-stiffness joints enhance interfacial shear resistance, effectively mitigating stress concentration effects at the rock mass-support interface induced by blasting shockwaves.

Specifically, under low joint stiffness conditions (< 10 GPa/m), localized interfacial slip is prone to occur under blasting loads, leading to shear plastic deformation in the superficial layer of the surrounding rock. As stiffness increases (> 15 GPa/m), the elastic modulus of the joints improves, enabling the transmission of impact energy as elastic waves into deeper rock strata, thereby reducing cumulative displacement in the superficial layer. Additionally, the regulatory role of stiffness in energy dissipation pathways cannot be overlooked. LS-DYNA dynamic finite element simulations reveal that when joint stiffness reaches 20 GPa/m, approximately 75% of blasting energy is dissipated through elastic deformation, while only 25% is converted into plastic deformation work—a ratio that is entirely reversed under low-stiffness conditions (5 GPa/m).

##### Nonlinear correlation mechanism between grouted joint stiffness and damage zone depth

The damage zone depth, a critical indicator for evaluating blasting-induced disturbance in surrounding rock, exhibits a significant nonlinear response relationship with grouted joint stiffness. Although specific numerical data for damage zone depths are incomplete, inverse analysis of the right wall deformation-stiffness curve (Fig. 14) and numerical simulations based on the Hoek-Brown rock mass strength criterion indicate that the quantitative relationship between damage depth (D) and joint stiffness (K) can be expressed as a piecewise function: For K < 10 GPa/m, D decreases exponentially with increasing K (D = 12.5e − 0.15 K), demonstrating substantial benefits of stiffness enhancement in suppressing damage propagation.These fitting relationships were empirically derived from the data points obtained through the series of numerical simulations presented in Fig. 14. The primary aim of this fitting is to quantitatively illustrate the discovered nonlinear trend and the transition point in the effectiveness of stiffness enhancement, rather than to provide a universally predictive model. The critical stiffness value (Kc) and the decay rates are specific to the geological conditions and model setup of this study.

When K > 15 GPa/m, the rate of D reduction decreases markedly, and the fitted curve approaches an asymptote (D ≈ 2.8–0.06 K), indicating a significant weakening of the marginal benefits of stiffness control. This nonlinear behavior is attributed to the propagation characteristics of blasting stress waves: Under low-stiffness conditions (K < 10 GPa/m), the joint interface fails to effectively reflect tensile waves, resulting in dense radial fractures within the surrounding rock and damage depths of 12–15 mm. When stiffness exceeds the critical threshold (Kc = 12 GPa/m), the reflectivity of stress waves at the joint interface increases to over 60%, localizing blasting energy in shallow rock layers and suppressing deep-seated damage.

This mechanistic understanding provides a theoretical basis for engineering optimization: For near-blast regions (< 2 m), joint stiffness should be elevated to at least 15 GPa/m to constrain damage depth within 5 mm; for distal regions (> 2 m), stiffness standards can be appropriately relaxed to 8–10 GPa/m to balance safety and material costs. It is critical to emphasize that the critical stiffness threshold (Kc) is modulated by the Geological Strength Index (GSI). When GSI < 50, Kc​ must be increased to 18 GPa/m to achieve equivalent damage control efficacy.

#### Grouting reinforcement of jointed rock masses

In deep rock excavation initiatives, the presence of joints frequently results in a high degree of fragmentation and substantial deformation of the surrounding rock. This diminishes the project’s applicability and longevity and poses significant threats to the safety of personnel and equipment. Grouting is recognized as an effective strategy for mitigating such risks. Joint grouting involves injecting a liquid with bonding properties into the rock’s joints; the slurry’s curing and cementing properties facilitate the bonding of the rock on either side of the joints, thereby enhancing the integrity and load-bearing capacity of the surrounding rock and effectively improving its safety and stability^34,35^. Extensive engineering case studies have demonstrated the joint grouting method’s effectiveness in achieving robust reinforcement outcomes^36^. The joint grouting technique has been validated by numerous engineering examples for its effectiveness in reinforcement.

Given that the strength and stiffness of joint filling materials are often significantly lower than those of the surrounding rock, the cementing effect of grouting can bolster the integrity of the weak filling body and improve the sealing between the filling body and the joint surfaces. To amplify the grouting effect, a certain liquid pressure is typically applied during the grouting process, ensuring that the slurry is more uniformly and thoroughly integrated with the filling. Thereafter, the slurry and the filler undergo a chemical reaction, resulting in a cement with enhanced strength, stiffness, and integrity. Additionally, the slurry forms “grouting rock bridges” and “grouting rock wedges” between the joint surfaces, as depicted in Fig. 14. The combined action of these three elements significantly strengthens and stiffens the joints, improves the stability of the surrounding rock, and safeguards the project’s security.

**Fig. 15:**
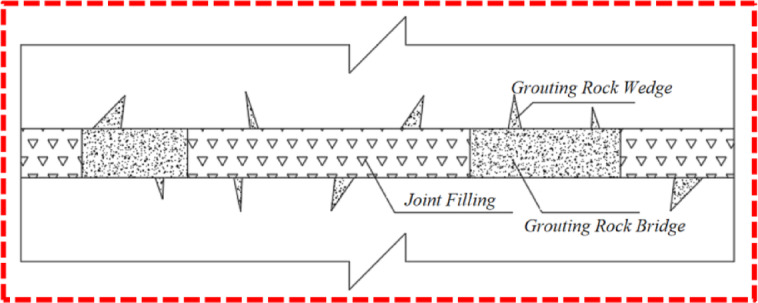
Schematic diagram of the principle of joint grouting reinforcement.

Based on the mechanical model of structural surface grouting consolidation body reinforcement, assuming that the cracks are deformed in both directions and the increase in positive stress is caused by the deformation of the extrusion-filled grouting material, the stiffness of the joints after grouting $\:{\text{K}}_{\text{n}}^{\prime}{\text{K}}_{\text{n}}^{\prime}$ can be expressed by the following Eq. 

6 $$\:{\text{K}}_{{\text{n}}}^{{\prime \,}} {\text{ = }}\frac{{{\text{1 + }}\beta }}{{{\text{cos}}^{{\text{2}}} \delta }}\left( {\alpha {\text{sin}}^{{\text{2}}} \delta \frac{{{\text{K}}_{{\text{n}}} {\text{ + K}}_{{{\text{ng}}}} }}{{{\text{K}}_{{\text{n}}} {\text{ + K}}_{{{\text{ng}}}} }}{\text{ + K}}_{{\text{n}}} } \right)$$

Where Kn is the initial joint stiffness before grouting; Kng is the equivalent stiffness of the grout-filled body; $\delta$ is the inclination angle of the structural surface; $\alpha$ is the grout filling rate; $\beta$ is the parameter related to the grouting material, which is taken to be in the range of 0.5 ~ 3.0 according to engineering experience.

In this project case, assuming that the F41 joint is strengthened by early grouting, due to the joint has the characteristics of good connectivity and high permeability, the single-point drilling and grouting method is adopted, the grouting material adopts ordinary cement monolithic slurry, and the grouting filling rate ${\alpha}{\alpha}$ is taken as 0.10, and $\beta$ is taken as 0.8. Then, according to the calculation of Eq. (6), we can get the stiffness of the joint after grouting $\:{\text{K}}_{\text{n}}^{\prime}{=7.26}\, \text{Kn}\text{=0.363}\text{E}$.

By adjusting the penalty function coefficients to enhance nodal stiffness within the numerical simulation, the simulation was reinitiated. The peak vibration velocity curves, as determined by the numerical simulation, were then compared with the velocities measured on the walls of both sides of the main variable cave before and after the nodal grouting, as illustrated in Fig. 15. The Fig. 15 indicates that the peak vibration velocities on both sides of the blast were reduced to some extent following joint grouting. Particularly on the right wall of the cave chamber, the reductions in vibration velocity in the XYZ directions were 3.05 cm/s, 2.86 cm/s, and 3.97 cm/s, respectively. Notably, the Z direction, which is horizontally perpendicular to the cave axis, experienced the most significant change, with an average reduction of 1.56 cm/s across the entire measurement line. On the left wall of the chamber, the amplitude decreased to a certain degree due to the increased integrity of the surrounding rock, though the relative decrease was less pronounced, with the maximum reduction being 0.79 cm/s in the Z direction.

**Fig. 16:**
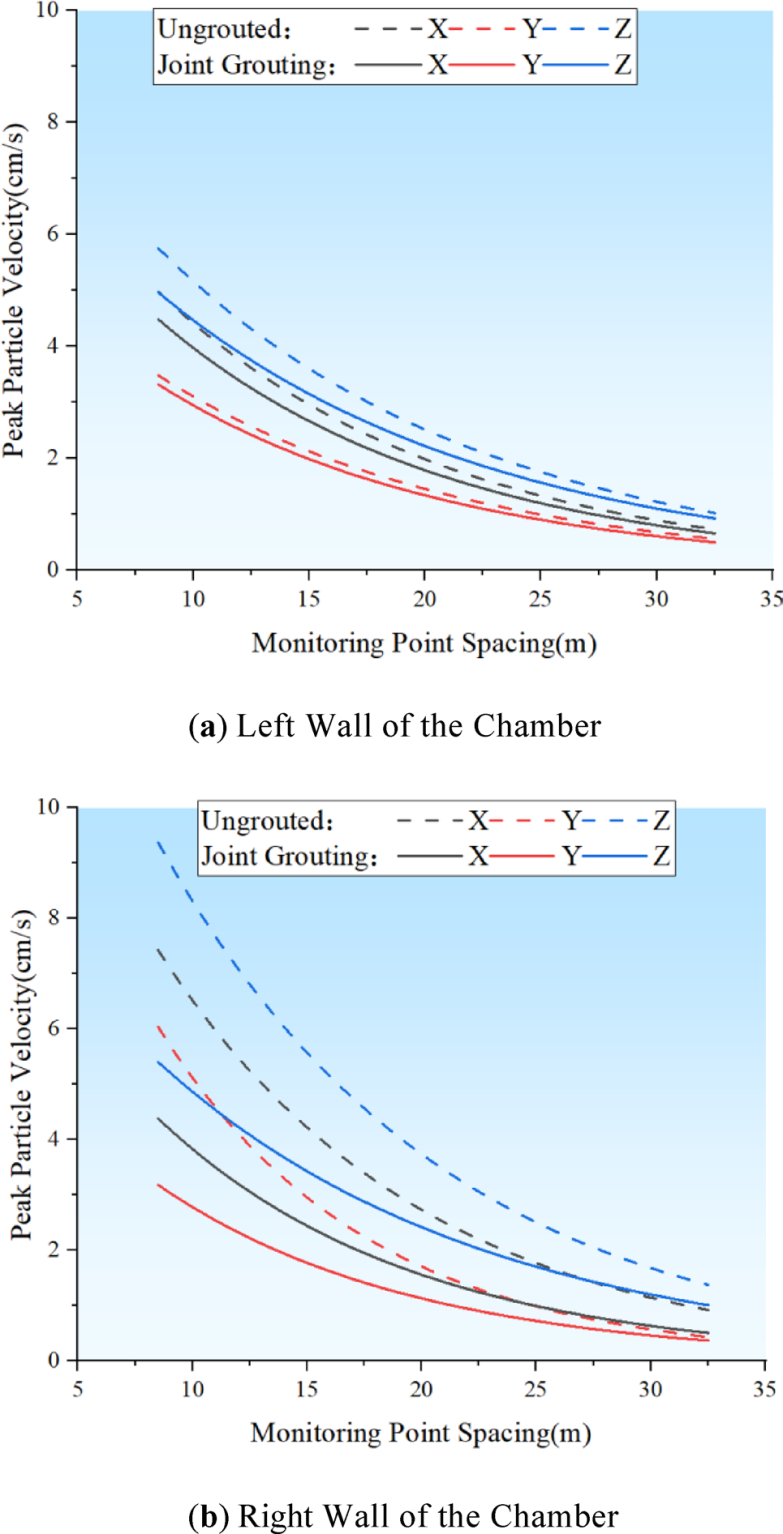
Comparison of peak blasting velocity values before and after section grouting.

The damage distribution cloud diagram post-joint grouting, as derived from numerical simulation, is depicted in Fig. 16. The Fig. 16 clearly demonstrates that the damage extent and depth of the surrounding rock on the right wall of the cavern chamber are markedly reduced, to the point of being scarcely distinguishable from that of the left wall. Additionally, the reflective damage zone previously observed in the F41 joints has vanished following joint grouting, indicating that joint grouting effectively controls the damage range of the surrounding rock.

**Fig. 17:**
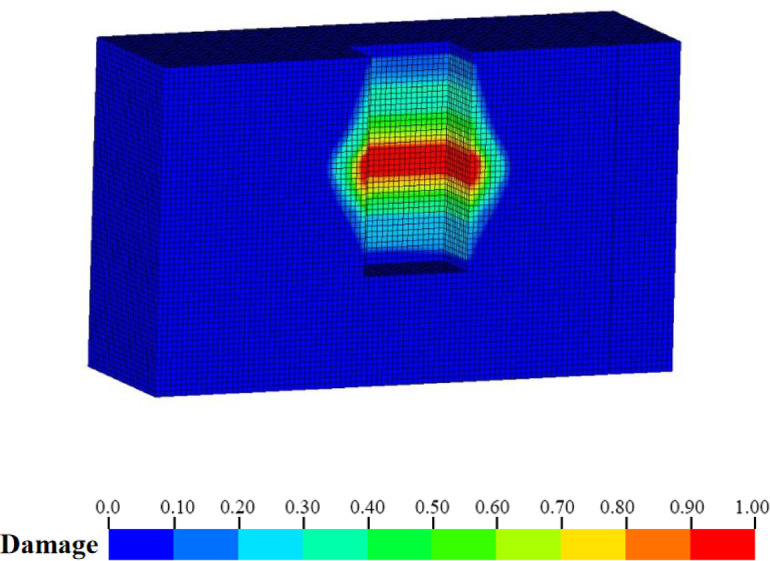
Cloud map of damage distribution after joint grouting.

**Fig. 18:**
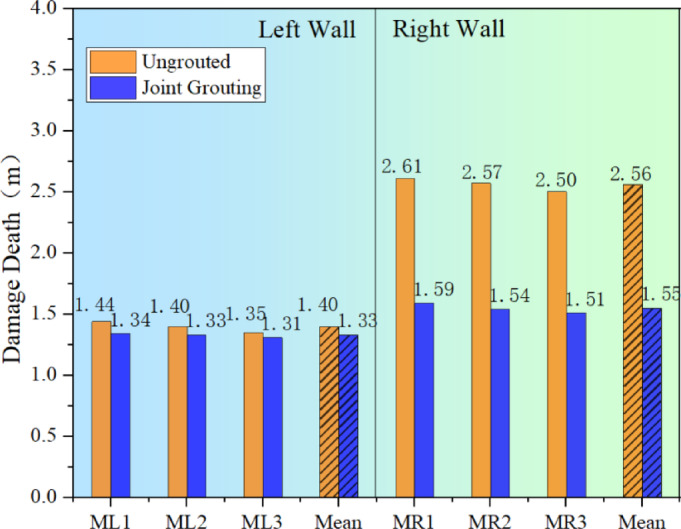
Comparison of damage depth after joint grouting.

Figure 17 presents a comparison of the damage depth post-joint grouting. It is evident from the Fig. 17 and the Fig. 18 that joint grouting can significantly reduce the damage depth in the surrounding rock, particularly on the right wall where the average damage depth has decreased by approximately 1.01 m, representing a reduction of about 40%. The control effect on the surrounding rock damage is highly noticeable. On the left wall, there is a minor reduction in the surrounding rock’s damage depth, from 1.40 m to 1.33 m, amounting to a total reduction of about 0.07 m. It can be concluded that joint grouting can indeed achieve the goal of controlling the damage to the surrounding rock, particularly in areas adjacent to the joints, with a very significant improvement effect .

Therefore, in the actual project, when the distance from the surrounding rock is closer to the existence of weak joints, can consider the use of advance drilling and grouting method, the joints play a reinforcing role, enhance the stiffness of the joints, increase the integrity of the surrounding rock, so as to achieve the control of blasting vibration and the scope of damage to the surrounding rock.

#### Fine blasting to reduce unloading rate

In addition to joint grouting, another widely adopted method in underground engineering is the practice of precision blasting. This method entails the strategic planning of borehole patterns and detonation sequences. By doing so, it seeks to alleviate the transient unloading effects of ground stress, thereby controlling the damage to surrounding rock caused by blasting excavation-induced disturbances. At the Waterfall Gully Hydropower Station’s underground plant, precision blasting is utilized in the floor and tailwater connection tunnel. By dividing the detonation process into segments and prolonging the duration from the initial ignition to the completion of blasting, this technique significantly reduces the disturbance and dynamic unloading stress related to blasting, ensuring excavation safety from a proactive control standpoint Proactive control measures are thus crucial for ensuring excavation safety.

**Fig. 19:**
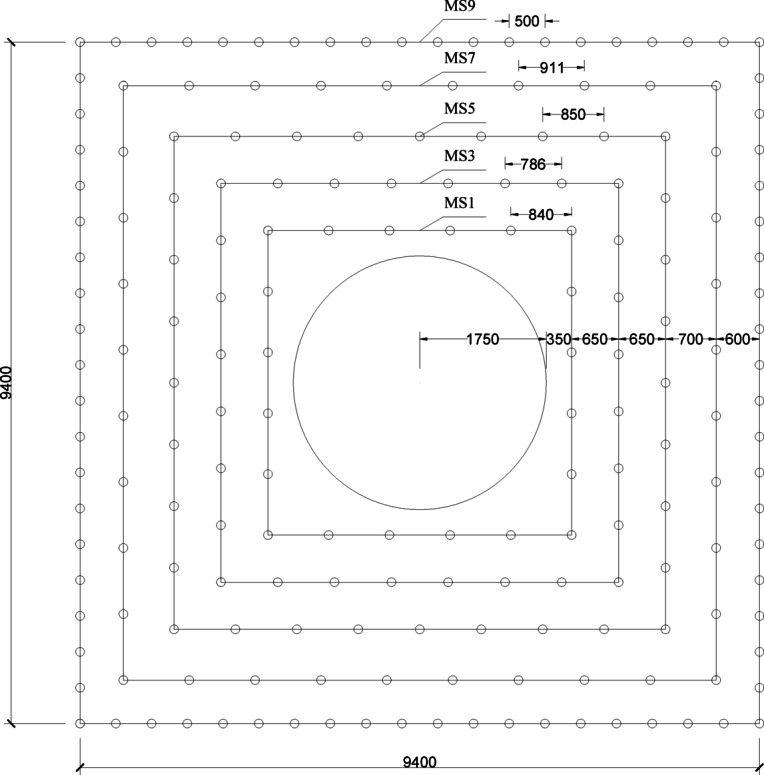
Optimized layout of blast holes for the secondary expansion blasting of the shaft.

In the case of this project, a similar approach is assumed to increase the number of drill holes and the number of gunnel sections, while reducing the amount of charge per hole. The distribution of optimised gun holes is shown in Fig. 16, and the drilling and charging parameters are shown in Table 3.

**Table 3:** Fine blasting drilling and charging Parameters.

Name of shell hole	Section of a detonator	Diameter of hole mm	Depth of hole cm	Orifice mm	Charge length cm	Single Hole Volume kg	Line density kg/m
Crumbling hole	MS1	42	350	840	280	1.37	0.4
	MS3	42	350	780	280	1.37	0.4
	MS5	42	350	650	280	1.37	0.4
	MS7	42	350	911	280	1.37	0.4
Peripheral hole	MS9	42	350	500	280	0.7	0.2

Numerical simulations were rerun to evaluate the changes in working conditions subsequent to the optimization of blasting parameters. The peak velocity curves, derived from numerical simulations on the walls of both sides of the main variable hole before and after optimization, are depicted in Fig. 19. In contrast to joint grouting, which predominantly reduces the vibration speed near the joint, the application of precision blasting to fine-tune blasting parameters has improved the blasting velocity on both sides of the wall. On the left wall, the maximum peak vibration velocities in the three directions were decreased by 1.85 cm/s, 1.53 cm/s, and 2.03 cm/s, respectively. On the right wall, the reduction in peak vibration velocities was marginally lower than that of joint grouting, with reductions in the three directions amounting to 2.53 cm/s, 2.53 cm/s, and 3.07 cm/s, respectively, equating to about 80% of the reduction achieved with joint grouting. This suggests that optimizing blasting parameters via precision blasting effectively lessens the overall dynamic effects of transient unloading and, in turn, indirectly reduces the blasting vibration velocity of the jointed rock mass.

**Fig. 20:**
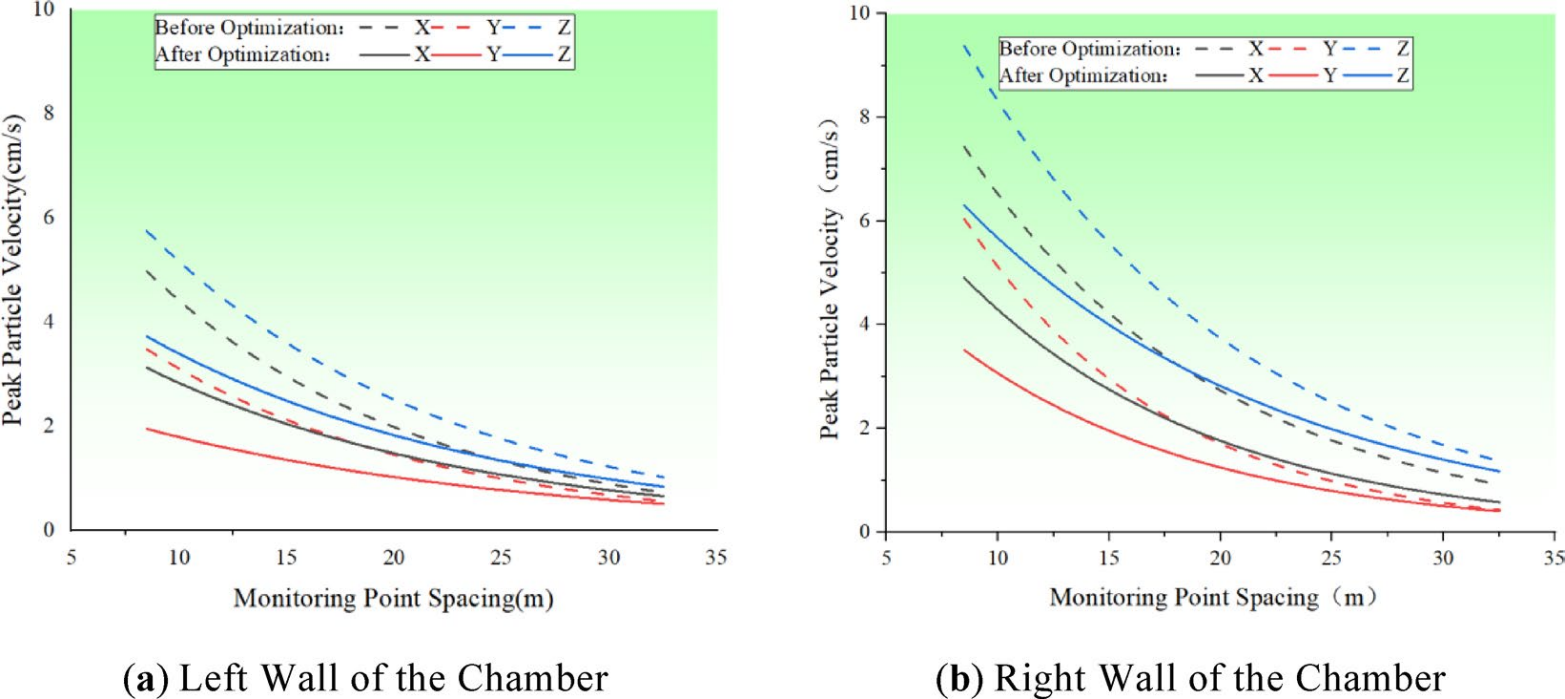
Comparison of blasting parameters before and after numerical optimisation of peak blasting velocity.

**Fig. 21:**
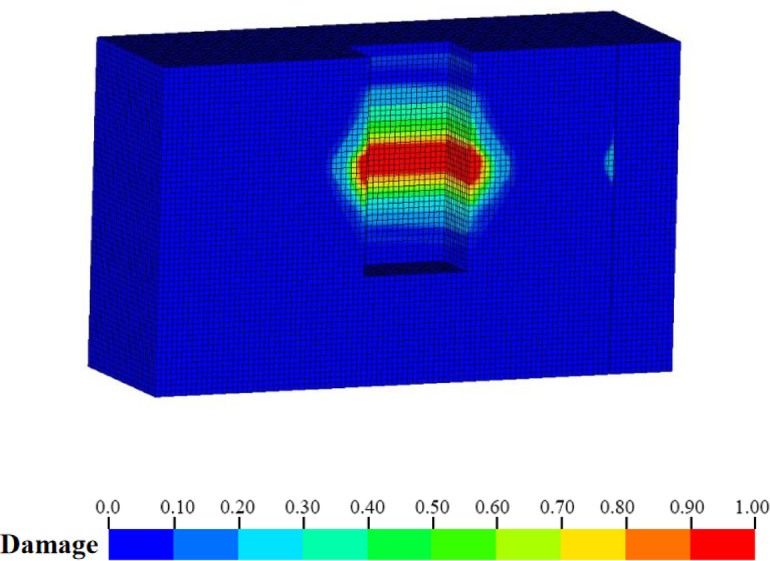
Damage distribution cloud after optimisation of blasting parameters.

The numerical simulation depicting the damage distribution subsequent to the implementation of fine blasting with optimized parameters is illustrated in the cloud diagram of Fig. 20. The Fig. 20 clearly shows a substantial reduction in both the damage extent and depth of the surrounding rock on both sides of the cavern chamber. However, while the reflective damage zone on the right side of the joints has reduced, it has not been completely eradicated.

**Fig. 22:**
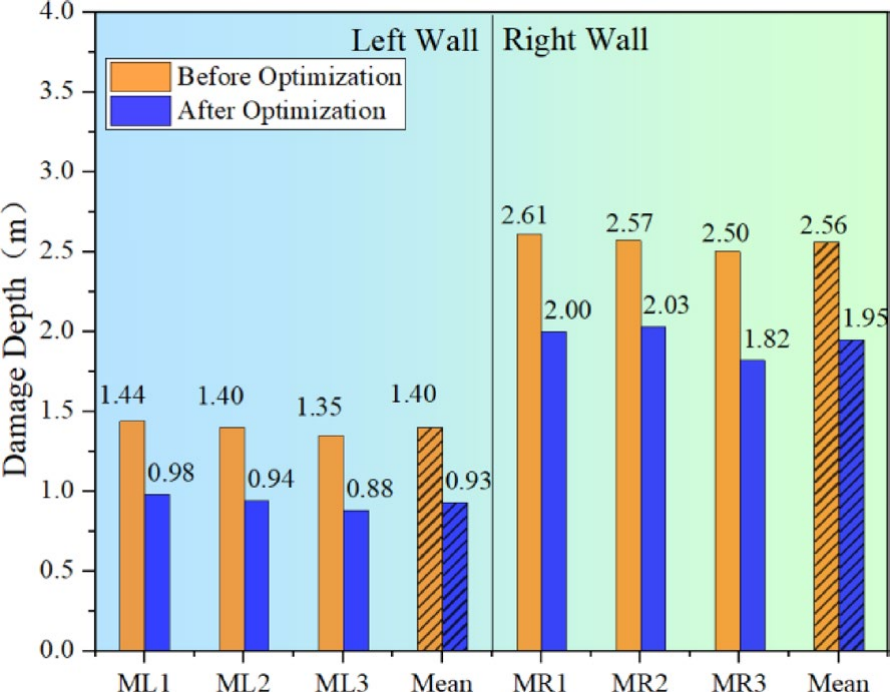
Comparison of damage depths for optimised blasting parameters.

Figure 21 displays a comparison of the damage depth following the optimization of blasting parameters via fine blasting. The Fig. 21 and the Fig. 22 indicates that by moderating the unloading rate through the optimization of blasting parameters, there is a significant reduction in the overall depth of damage within the surrounding rock. The mean damage depth on both sides of the chamber decreased by 0.47 m and 0.61 m, respectively. Compared to the joint grouting method, the enhancement in damage depth on the left side is more pronounced, while the improvement on the right side is relatively less marked.

Thus, in the context of practical project implementation, when the objective is to control and mitigate the extent of damage to the surrounding rock, in addition to employing the section grouting method, one can also contemplate the use of precision blasting techniques. By prolonging the unloading time from the initial detonation to the conclusion of blasting, the unloading rate is diminished, which in turn effectively reduces the disturbance caused by blasting and the dynamic unloading of geostatic stress. This strategy, aimed at minimizing the overall dynamic response of the surrounding rock, is crucial for ensuring the safety of excavation works.

## Conclusion

The economic feasibility of the proposed measures, particularly grouting, must be considered. Grouting incurs additional costs for materials, equipment, and time, making it more suitable for large-scale critical projects (e.g., major underground powerhouses) with high stability requirements. For general projects, priority should be given to cost-effective precision blasting optimization or localized grouting in key areas. A cost-benefit analysis is crucial for decision-making.

The findings of this study are most directly applicable to deep, hard rock masses(e.g., granite, basalt)under high in-situ stress containing weak joints. When generalizing to other geological conditions, attention must be paid to: (1)joint properties(infilling, orientation, spacing, stiffness); (2) in-situ stress levels–control effects are more pronounced under higher stress; and(3)rock type–damage mechanisms and control strategies in soft rock may differ significantly.

1. On-site measurements of blasting vibration velocity and acoustic damage depth have demonstrated that the presence of joints within the rock mass significantly increases the peak blasting vibration velocity and damage depth due to the reflective effect of these joints. Notably, on the right wall of the chamber, which included joints, the peak vibration velocity rose by approximately 3.55 cm/s, and the damage depth increased by about 1.0 m, in contrast to the left side without joints.
2. In the case of deep shafts with joints and fissures, joint grouting is an effective method for controlling damage to the surrounding rock, especially in areas near the joints where the improvement is considerably significant. After grouting, the peak vibration velocity of the chamber’s walls during blasting is reduced to some degree, with the right wall of the chamber exhibiting reductions of 3.05 cm/s, 2.86 cm/s, and 3.97 cm/s in three-directional vibration velocities. Regarding damage, joint grouting effectively decreases the damage depth in the surrounding rock; the right wall of the chamber saw a decrease of about 1.01 m, equating to a reduction of approximately 40%.
3. Compared to joint grouting, the optimized blasting parameter scheme is even more effective in reducing both the blasting vibration velocity and the depth of damage to the perimeter rock on both sides of the cave wall. The maximum peak vibration speeds in the three directions were decreased by 2.53 cm/s, 2.53 cm/s, and 3.07 cm/s, respectively, and on the non-jointed side of the wall, the reductions were 1.85 cm/s, 1.53 cm/s, and 2.03 cm/s, respectively. The average damage depths were reduced by 0.47 m on the left wall and 0.61 m on the right wall, with improvements being more pronounced on the left side. This method indirectly mitigates the blast vibration rate and damage extent by lessening the dynamic impact of transient unloading.
4. Engineering applicability and adaptability: The proposed methodology forms a comprehensive control system. Its application across different geological conditions is recommended as follows:

Hard, dense rock (e.g.,basalt): Direct application is effective. Soft/fractured strata(e.g.,shale): Requires reduced charge per hole and modified grout viscosity.High-permeability rock(e.g.,fractured sandstone): Employs rapid-setting grouts and optimized borehole spacing.Layered/anisotropic rock (e.g.,slate): Necessitates directional grouting and drilling aligned with bedding planes.High-stress environments: Benefits from integrated staged excavation and stress-relief techniques.This system should be dynamically optimized using integrated geophysical detection and numerical simulation.

## Data Availability

The data that support the findings of this study are available from the corresponding author, [Song], upon reasonable request.
